# Pleiotropic *Clostridioides difficile* Cyclophilin PpiB Controls Cysteine-Tolerance, Toxin Production, the Central Metabolism and Multiple Stress Responses

**DOI:** 10.3389/fphar.2019.00340

**Published:** 2019-04-05

**Authors:** Can Murat Ünal, Mustafa Safa Karagöz, Mareike Berges, Christina Priebe, José Manuel Borrero de Acuña, Josef Wissing, Lothar Jänsch, Dieter Jahn, Michael Steinert

**Affiliations:** ^1^Institut für Mikrobiologie, Technische Universität Braunschweig, Braunschweig, Germany; ^2^Moleküler Biyoteknoloji Bölümü, Türk-Alman Üniversitesi, Istanbul, Turkey; ^3^Braunschweig Integrated Centre of Systems Biology, Braunschweig, Germany; ^4^Cellular Proteomics Research, Helmholtz Centre for Infection Research, Braunschweig, Germany; ^5^Helmholtz Centre for Infection Research, Braunschweig, Germany

**Keywords:** *Clostridium difficile*, peptidyl-prolyl-*cis/trans*-isomerase (PPIase), cytotoxicity, interactomics, transcription

## Abstract

The Gram-positive pathogen *Clostridioides difficile* is the main bacterial agent of nosocomial antibiotic associated diarrhea. Bacterial peptidyl-prolyl-*cis/trans*-isomerases (PPIases) are well established modulators of virulence that influence the outcome of human pathologies during infections. Here, we present the first interactomic network of the sole cyclophilin-type PPIase of *C. difficile* (CdPpiB) and show that it has diverse interaction partners including major enzymes of the amino acid-dependent energy (LdhA, EtfAB, Had, Acd) and the glucose-derived (Fba, GapA, Pfo, Pyk, Pyc) central metabolism. Proteins of the general (UspA), oxidative (Rbr1,2,3, Dsr), alkaline (YloU, YphY) and cold shock (CspB) response were found bound to CdPpiB. The transcriptional (Lrp), translational (InfC, RFF) and folding (GroS, DnaK) control proteins were also found attached. For a crucial enzyme of cysteine metabolism, *O*-acetylserine sulfhydrylase (CysK), the global transcription regulator Lrp and the flagellar subunit FliC, these interactions were independently confirmed using a bacterial two hybrid system. The active site residues F50, F109, and F110 of CdPpiB were shown to be important for the interaction with the residue P87 of Lrp. CysK activity after heat denaturation was restored by interaction with CdPpiB. In accordance, tolerance toward cell wall stress caused by the exposure to amoxicillin was reduced. In the absence of CdPpiB, *C. difficile* was more susceptible toward L-cysteine. At the same time, the cysteine-mediated suppression of toxin production ceased resulting in higher cytotoxicity. In summary, the cyclophilin-type PPIase of *C. difficile* (CdPpiB) coordinates major cellular processes via its interaction with major regulators of transcription, translation, protein folding, stress response and the central metabolism.

## Introduction

The multi-resistant Gram-positive obligate anaerobe *Clostridioides (Clostridium) difficile* is the main nosocomial bacterial agent of antibiotic treatment associated diarrhea (Hopkins and Wilson, 2018). The rise of *C. difficile* infection (CDI) coincides with the introduction of clindamycin, the first broad-band lincosamin against anaerobic Gram-negative pathogens (Bartlett et al., 1977; Lusk et al., 1978). Currently, first line of treatment is the use of antibiotics like vancomycin, metronidazole and fidaxomicin. However, high recurrence rates, especially after treatment with vancomycin or metronidazole, make this disease difficult to manage (Mullane, 2014; Hopkins and Wilson, 2018).

A major factor influencing the course and outcome of CDI is the composition of the host intestinal microbiome and, connected to it, the bile acid status of the infected individual. Patients undergoing a CDI typically have an antibiotic dependent change of their microbiome followed by a transition from antibacterial to less antibacterial bile acids in their gastrointestinal tract (Theriot et al., 2016). Most commonly, CDI manifests in form of pseudomembranous colitis, which is a strong inflammation of the large intestine. This is caused by massive tissue destruction and leukocytosis due to the production of two large glycosylating toxins, TcdA and TcdB. These exert their cytotoxic activity by inactivating small GTPases and disturbing the actin cytoskeleton dynamics leading to disruption of the gut epithelial barrier (Barbut et al., 2007; Rupnik et al., 2009; Chandrasekaran and Lacy, 2017).

The production of TcdA and TcdB typically coincides with the entry of the bacteria into the stationary phase. Nevertheless, it is controlled by complex regulatory processes on multiple levels and by diverse environmental cues, including subinhibitory concentrations of antibiotics, temperature or the availability of carbon sources and amino acids (Dupuy and Sonenshein, 1998; Bouillaut et al., 2015). One central metabolite in this respect is L-cysteine that represses toxin production (Karlsson et al., 2000). Apart from that, L-cysteine also affects the expression of genes of amino acid biosynthesis, fermentation, energy metabolism, iron acquisition and stress response (Dubois et al., 2016; Gu et al., 2018). Furthermore, there is a strong interconnectedness between iron and L-cysteine dependent gene regulation circuits. Several of the genes of the latter contain Fur boxes in their promoters, which are binding sites for the global iron-responsive transcriptional ferric uptake regulator (Fur) (Dubois et al., 2016). If not metabolized effectively L-cysteine also has an inhibitory effect on growth on *C. difficile* as it was nicely shown in case of a mutant lacking the cysteine desulfidase CdsB (Gu et al., 2017).

Although there is strong correlation between CDI severity and the capability of the bacteria to produce TcdA and TcdB, this alone does not explain the virulence spectrum of *C. difficile* in its entirety. Accordingly, several other virulence factors such as extracellular proteases, surface layer proteins, a fibronectin binding protein (Fbp68), a collagen binding protein (CbpA), type IV pili and flagella have been shown or are suspected to contribute to disease severity and host colonization (Geric et al., 2004; Janoir, 2016; Ünal and Steinert, 2016; Péchiné et al., 2018). Especially, the regulation of flagellation and its main structural component flagellin (FliC) is crucial for modulating adherence to host cells, promoting colonization and eliciting immune responses via toll-like receptor 5 (Tasteyre et al., 2001; Dingle et al., 2011; Batah et al., 2016). Moreover, flagellation and toxin production are co-regulated by an intricate genetic switch adding up to the complexity of toxin-related pathogenicity of *C. difficile* (Aubry et al., 2012; Anjuwon-Foster and Tamayo, 2017).

Currently, many single components and mechanisms of CDI that contribute to host colonization, disease outbreak and dissemination are known. However, their concerted action is still not fully understood. Bacterial PPIases (FKBPs, parvulins and cyclophilins) have in many instances been shown to modulate infectious processes (Ünal and Steinert, 2014). Among those are prominent representatives like Mip and Mip-like FKBPs, or parvulins, like SurA in Gram-negative as well as PrsA and PrsA2 in Gram-positive bacteria (Jacobs et al., 1993; Heikkinen et al., 2009; Behrens-Kneip, 2010; Alonzo et al., 2011; Obi et al., 2011; Ikolo et al., 2015; Ünal and Steinert, 2015). Additionally, recent studies have shown the participation of cyclophilins in the maturation of a secreted nuclease in *Staphylococcus aureus* as well as in virulence traits, like biofilm formation in *Escherichia coli* or in sliding motility and amoeba infection by *Legionella pneumophila* (Skagia et al., 2016; Wiemels et al., 2017; Rasch et al., 2018). The aim of our study was to identify the interaction partners of the sole cyclophilin of *C. difficile* (CdPpiB) and to characterize its contribution to bacterial physiology as well as virulence. Our results show an unprecedented relationship between bacterial cysteine metabolism and CdPpiB with far reaching implications on virulence. Furthermore, a protein network controlling major cellular function including transcription, translation, protein folding, metabolism, and stress responses was uncovered.

## Materials and Methods

### Bacterial Strains and Culture

Bacterial strains and plasmids used in this study are listed in Table 1. *C. difficile* 630*Δerm* (hereafter referred to as wild type or wt) and its derivatives were cultured in BHIS medium (brain-heart infusion broth (Carl Roth GmbH, Germany) supplemented with 5 g/L yeast extract (BD Bacto^TM^, United States) and 1 g/L L-cysteine (Sigma-Aldrich^^®^^, Germany) or TY-medium (30 g/L tryptone, 20 g/L yeast extract) under anaerobic conditions (95% N_2_/5% H_2_). If necessary, 5 μg/mL erythromycin or 15 μg/mL thiamphenicol were added. *Escherichia coli* and *Bacillus megaterium* were cultured in LB medium supplemented with 100 μg/mL ampicillin, 10 μg/mL tetracycline or 30 μg/ml chloramphenicol at 37°C and 200 rpm. All antibiotics were purchased from Sigma-Aldrich^^®^^. Media were solidified by adding 1.5% (wt/vol) agar when needed.

**Table 1:** Bacterial strains and plasmids used in this study.

Name	Features	References
*C. difficile* str. 630Δ*erm*	Erythromycin-sensitive derivative of strain 630	DSMZ^†^ (DSM 28645) (Hussain et al., 2005)
*C. difficile* str. 630Δ*erm*Δ*ppiB*	*cd03310::ClosTron*, Erm^R^	This study
*E. coli* DH10β	Strain for cloning and plasmid propagation, F^−^ *mcrA Δ*(*mrr*-*hsdRMS*-*mcrBC*) Φ80d*lacZ*Δ*M15* Δ*lacX74 endA1 recA1 deo*R Δ(*ara*, *leu*)7697 *araD*139 *galU galK nupG rpsL* λ^−^	Grant et al., 1990
*E*. *coli* CA434	*E. coli* HB101 carrying the R702- conjugative plasmid (Tra^+^, Mob^+^)	Purdy et al., 2002
*E. coli* BTH101	F^−^, *cya*-99, *araD*139, *galE15*, *galK16*, *rpsL1* (Str^R^), *hsdR2*, *mcrA1*, *mcrB1*	Euromedex, France
*B. megaterium* MS941	Strain for recombinant production, deficient of the major secreted protease, Δ*nrpM*	Wittchen and Meinhardt, 1995
pMTL82151	Shuttle vector for complementation of *C. difficile* mutants; pBP1-*ori* (Gram-pos.), ColE1 *ori* (Gram-neg.), *tra*, MCS, Cm^R^	Heap et al., 2009
pPpiB_NStrep	pMTL82151 carrying wild type *ppiB* including its 250 bp upstream and 50 bp downstream regions and a N-terminal StrepII-tag	This study
pDSW1728	P_tet_::*mCherryOpt*, Cm^R^	Ransom et al., 2015
pPpiB-tet	*mCherryOpt* in pDSW1728 replaced by *cd03310* by SacI and BamHI yielding P*_tet_*::*ppiB*, Cm^R^	This study
pN-STREPXa1622	Vector for recombinant protein production in *B. megaterium*, P*_xylA_*-StrepII-Xa-MCS, Tc^R^, Amp^R^	Biedendieck et al., 2007
p119_PpiB	pN-STREPXa1622 in which the ORF of *ppiB* (CD630_03310) was cloned using BglII and SphI	This study
p119_CysK	pN-STREPXa1622 in which the ORF of *cysK* (CD630_15940) was cloned using BglII and SphI	This study
pKNT25	Low copy plasmid carrying the T25 subunit of adenylate cyclase with N-terminal MCS, Km^R^	Euromedex, France
pKNTB	pKNT25 carrying wt *ppiB*	This study
pUT18	High copy plasmid carrying the T18 subunit of adenylate cyclase with N-terminal MCS, Amp^R^	Euromedex, France
pUT18_CysK	pUT18 carrying wt *cysK* of *C. difficile* str. 630Δ*erm*	This study
pUT18_Lrp	pUT18 carrying wt *lrp* of *C. difficile* str. 630Δ*erm*	This study
pUT18_FliC	pUT18 carrying wt *fliC* of *C. difficile* str. 630Δ*erm*	This study

*^†^Deutsche Sammlung von Mikroorganismen und Zellkulturen GmbH.*

### *In vivo* Cross-Linking for the Identification of PpiB Interaction Partners

Possible interaction partners of PpiB were identified by *in vivo* cross-linking as previously described (Borrero-de Acuña et al., 2015). Briefly, *C. difficile* str. 630Δ*erm*Δ*ppiB* + PpiB-NStrep was grown for 8 h in 25 mL of BHIS and refreshed 1:1000 in 2 L BHIS. After 16 h 0.125 % (vol/vol) formaldehyde (AppliChem, Germany) was added for chemical cross-linking (30 min at 37°C). Excess formaldehyde was quenched for 5 min at 37°C by the addition of glycine at a final concentration of 130 mM. Cells were harvested and washed twice with PBS (4000 *g* for 20 min at 4°C). Finally, the cells were resuspended in 25 mL PBS supplemented with complete Protease Inhibitor Cocktail (Roche, Switzerland), and homogenized using FRENCH^^®^^ Press (Thermo, United States) or glass beads and FastPrep^^®^^ (MP Biomedicals, United States). Cell debris was removed by centrifugation (10,000 *g*, 10 min, 4°C), and filtering the supernatant through a 0.45 μm syringe filter. The supernatant was applied onto a 3 mL Strep-Tactin^^®^^ column (iba Lifesciences, Germany) in order to purify PpiB and its cross-linked partners according to manual instructions. The elution fraction was concentrated using Amicon Ultra-0.5 mL centrifugal filters (Merck, Germany) with a molecular cut-off of 10 kDa. For analysis, the concentrated eluate was mixed with 4x SDS loading buffer and subjected to decrosslinking by heating at 95°C for 30 min. Proteins were separated on a 12% acrylamide gel, and stained using Coomassie stain [100 g/L (NH_4_)_2_SO_4_, 100 mL/L H_3_PO_4_, 20% vol/vol methanol]. Bands of interest were analyzed by LC-MS as previously described (Borrero-de Acuña et al., 2016). Resulting protein identifications (hits) were filtered according to their overall coverage by the LC/MS approach and the overall number of unique peptides. Hits with at least 15% coverage and two or more unique peptides were taken into account as this indicated robust enrichment. Ribosomal proteins were discarded because of their intrinsically high abundance in the cell and their known unspecific protein-protein interactions due to their overall strong basic character.

### Bacterial Two Hybrid (BACTH) Screening

Protein-protein interactions were confirmed using the BACTH-system of Euromedex (France), which bases on an adenylate cyclase (CyaA) dependent reporter system. For this, *ppiB*, *fliC* (CD630_02390), *lrp* (CD630_35440) and *cysK* (CD630_15940) were cloned in frame with in two different vectors, each containing one subunit of the adenylate cyclase of *Bordetella pertussis* (Tables 1, 2). After confirmation of correct cloning, the CyaA-deficient strain *E. coli* BTH101 was transformed with combinations of plasmids carrying *ppiB* and the respective interaction partners, and transformants were selected on LB-agar containing 50 μg/mL kanamycin and 100 μg/mL ampicillin. Single transformants were then streaked out on MacConkey-agar (BD Difco^TM^, United States) supplemented with 50 μg/mL kanamycin and 100 μg/mL ampicillin, incubated for 2 days at 30°C, and checked for pink coloring due to CyaA-activity.

### Measuring the Interaction of PpiB and Lrp and Their Amino Acid Substitution Mutants by β-Galactosidase Assay

*Escherichia coli* BTH101 carrying plasmid combinations that had been confirmed on MacConkey-agar were used in a β-galactosidase assay for quantitative comparison of interactions between amino acid mutants of PpiB and Lrp, respectively. For this, at least six clones of each transformation were picked and grown o/n (30°C, 200 rpm) in 5 mL LB medium supplemented with 50 μg/mL kanamycin, 100 μg/mL ampicillin and 0.5 mM IPTG (GERBU, Germany). Prior to the assay, bacterial growth was stalled by chilling the cultures for at least 30 min at 4°C and the OD_600_ was determined. Bacteria of 200 μL culture were pelleted (3500 rpm, 5 min, 4°C), and resuspended in 900 μL of Z-Buffer (60 mM Na_2_HPO_4_, 40 mM NaH_2_PO_4_, 10 mM KCl, 1 mM MgSO_4_, and freshly added 50 mM β-mercaptoethanol, pH 7.0). Bacteria were lysed by the addition of 50 μL chloroform and 50 μL 0.1% SDS (wt/vol) for 5 min at 30°C. After this, 200 μl of *o*-nitrophenyl-β-D-galactoside (ONPG, Roth GmbH, Germany) dissolved at 4 mg/mL in phosphate buffer (60 mM Na_2_HPO_4_, 40 mM NaH_2_PO_4_, pH 7.0) was added. The reactions were kept for 12 min at 30°C and subsequently stopped by the addition of 500 μL 1 M Na_2_CO_3_. After removal of cell debris and chloroform by centrifugation (14000 rpm, 5 min, 4°C) absorption at 420 and 550 nm were recorded with a VarioSkan^TM^ (Thermo Fisher, United States) plate reader. β-galactosidase activity, expressed in Miller Units, was calculated according to the following formula:

$$\mathrm{Miller Units}=\frac{1000\times (\text{OD}420-1.75\times \text{OD}550)}{t[\min]\times V[\text{mL}]\times \text{OD}600}$$

### Construction of a *ppiB* Destruction Mutant (Δ*ppiB*) in *C. difficile* 630Δ*erm* Using ClosTron

A synthetic vector containing the region of *ppiB* (CD630_03310/CDIF630erm_00459) was designed with the help of the ClosTron website^1^ using the Perutka algorithm (Perutka et al., 2004). *E*. *coli* CA434 was transformed with customized vectors for mating with *C. difficile* 630*Δerm* cells as described previously (Heap et al., 2009, 2010). Mutants were selected on BHIS containing 15 μg/mL erythromycin and confirmed by PCR and sequencing using gene specific primers (Table 2).

**Table 2:** Primers used in this study.

Name	Sequence^†^	Features	References
PpiB_KompF1	gatgagcttGAACCATTAATTGGGGATAATG	HindIII, for cloning into pMTL82151	This study
PpiB_KompR1	atcatgcggccgcTTTATCCTCATTTTTAAGTA ATATATA	NotI, for cloning into pMTL82151	This study
PpiB_NStrep_F	cgggtggctccaTTCCATAATTTATTACCTTCCTTTTC	For introducing N-terminal SrepII-Tag	This study
PpiB_NStrep_R	cagtttgaaaaaAATAAAAATCCTATAGTAACTATAGAAATG	For introducing N-terminal SrepII-Tag	This study
PpiB_For4	TCTgagctcATGAAAAGGAAGGTAATAAATTATG	SacI, for cloning into pDSW1728	This study
PpiB_Rev4	ATAggatccTTAGTTTTTTTCTACATCTGAGTAA	BamHI, for cloning into pDSW1728	This study
CdPpiB_TH_For	gactctagagGAAAATAAAAATCCTATAGTAAC	XbaI, for cloning into BACTH vectors	This study
CdPpiB_TH_Rev	ctcggtaccgcGTTTTTTTCTACATCTGAGTAA	KpnI, for cloning into BACTH vectors	This study
CdLrp_TH_For	gactctagagGATGTTACAGATTACAGAATC	XbaI, for cloning into BACTH vectors	This study
CdLrp_TH_Rev	ctcggtaccgcCAATATTGATTTTGCTTGTATTG	KpnI, for cloning into BACTH vectors	This study
CdFliC_TH_For	gactctagagAGAGTTAATACAAATGTAAGTG	XbaI, for cloning into BACTH vectors	This study
CdFliC_TH_Rev	ctcggtaccgcTCCTAATAATTGTAAAACTCC	KpnI, for cloning into BACTH vectors	This study
CdCysK_TH_For	gactctagagTTATATAATAACGCATTAGAGTT	XbaI, for cloning into BACTH vectors	This study
CdCysK_TH_Rev	ctcggtaccgcAAATATTCCCATAGACATATAC	KpnI, for cloning into BACTH vectors	This study
Lrp_P32A_invF	GCAGTTTCAGAAAGAGTCAAAAG		This study
Lrp_P32A_invR	tgcAGAAGTTAAACCAACTATTTTTCC		This study
Lrp_P56A_invF	GATTCATTAGGCAGAGTTATAAAG		This study
Lrp_P56A_invR	tgcGTTGACAATAGCTTTATATCCTTC		This study
Lrp_P72A_invF	gcaAGCAATGGATATACAGAATTTATTG		This study
Lrp_P72A_invR	AAGAGAAATATGAATAAATGCCTTTAT		This study
Lrp_P87A_invF	AGGATTGTAGAATGTCACCATAT		This study
Lrp_P87A_invR	tgcGTCCTTTGCAGCTGACTC		This study
Lrp_P135A_invF	gcaATACAAGCAAAATCAATATTGGC		This study
Lrp_P135A_invR	CGTTGATAGTATAACAGAGG		This study
PpiB_R50A_invF	GTAATACCAGGATTTATGATAC		This study
PpiB_R50A_invR	tgcATGAAATATTATTCCATTGTAATATC		This study
PpiB_F109A_invF	gcaTTCATAATGCATAAAAACTCACCAC		This study
PpiB_F109A_invR	TTGAGAACCAGCTGAATTAGGTG		This study
PpiB_F110A_invF	gcaATAATGCATAAAAACTCACCAC		This study
PpiB_F110A_invR	AAATTGAGAACCAGCTGAATTAG		This study
PpiB_For3	atgagatcttaATGGAAAATAAAAATCCTATAGTAAC	BglII, for cloning into pN-STREPXa1622	This study
PpiB_Rev3	tagcatgcTTAGTTTTTTTCTACATCTGAGTAAT	SphI, for cloning into pN-STREPXa1622	This study
CdCysK_119_F1	gccagatctTGTTATATAATAACGCATTAGAG	BglII, for cloning into pN-STREPXa1622	This study
CdCysK_119_R1	ccggcatgcTTAAAATATTCCCATAGACATATAC	SphI, for cloning into pN-STREPXa1622	This study
EBS universal primer	CGAAATTAGAAACTTGCGTTCAGTAAAC		Heap et al., 2007
ErmRAM_F	ACGCGTTATATTGATAAAAATAATAGTGGG		Heap et al., 2007
ErmRAM_F	ACGCGTGCGACTCATAGAATTATTTCCTCCCG		Heap et al., 2007

*^†^Restriction recognition sites are underlined; bases for the respective alanine exchange are highlighted.*

### Assessing Growth and Susceptibility Toward Amoxicillin

Bacterial growth was monitored by refreshing cultures of *C. difficile* grown overnight (o/n) in 25 mL medium to a starting optical density (OD_600__nm_) of 0.05 in 25 mL fresh medium. Increase in OD_600__nm_ was measured starting with t_0_ and every 60 min until the cultures reached stationary phase. For assessing amoxicillin susceptibility, o/n cultures of wild type *C. difficile* and its Δ*ppiB* mutant were prepared in 20 mL BHIS medium. The next morning the cultures were adjusted to an OD_600__nm_ of 0.1 in BHIS medium. Amoxicillin stock solution was prepared fresh (2 mM in ddH_2_O) and a 1:1 serial dilution starting at 256 μM was prepared in 100 μl of BHIS distributed in the wells of a 96-well plate. Following this, 100 μL of the bacterial suspension were added into each well resulting in a final OD of 0.05. Bacteria were grown for 6 h at 37°C under anaerobic conditions, and the final OD was measured at 600 nm using a VarioSkan^TM^ (Thermo Fisher, United States) plate reader.

### Complementation of *C. difficile*Δ*ppiB* With StrepII-Tagged PpiB and Implementing a Tetracycline- Inducible Promoter

In order to achieve a complementation of the mutant with wild type PpiB carrying a N-terminal StrepII-tag for affinity purification, *ppiB* was amplified including its 250 bp up- and 100 bp-downstream regions using the primer combination PpiB_KompF1/R1. This fragment was cloned into the shuttle vector pMTL82151 with *HindIII* and *BamHI* yielding pMTL_ppiB. This plasmid was used as a template for an inverse PCR by which an N-terminal StrepII-tag was added to PpiB with the primer combination PpiB_NStrep_F/R. The resulting PCR-product was gel purified, phosphorylated with T4 polynucleotide kinase (NEB) and re-ligated with T4 ligase (NEB) according to manufacturer’s instructions. Plasmids carrying the desired mutations were selected in *E. coli* DH10β and verified by sequencing. For complementation of the Δ*ppiB* mutant under the control of a tetracycline-inducible promoter (P_tet_), the ORF of CdPpiB was cloned into pDSW1728 utilizing the restriction sites *SacI* and *BamHI*. The Δ*ppiB* mutant was transformed by conjugation and selected using 15 μg/ml thiamphenicol. For complementation studies the bacteria were cultured with 10 ng/mL anhydrotetracycline (AT), and a dose-dependent complementation was achieved by performing the assays with decreasing concentrations of TA starting at 400 ng/mL.

### Recombinant Protein Production

For recombinant production, *ppiB* or *cysK* were amplified with Q5-Polymerase (NEB GmbH, Germany) and cloned into the production vector pN-STREPXa1622 using the restriction sites BglII and SphI yielding N-terminally StrepII-tagged proteins (Biedendieck et al., 2007). For recombinant protein production, an o/n culture of transformed *B. megaterium* MS941 was prepared, and the next morning refreshed 1:100 in 300 ml LB medium containing 10 μg/mL tetracycline (Biedendieck et al., 2011). The cells were induced at an OD_600__nm_ of ∼0.4 by the addition of D-xylose (Carl Roth GmbH, Germany) at a final concentration of 0.5 % (w/v). After 6 h, the cells were harvested (3000 *g*, 15 min, 4°C), and the pellet was once washed with 1x PBS (5000 *g*, 15 min, 4°C). Cells were resuspended in 25 mL ddH_2_O, and disrupted by FRENCH^^®^^ Press (Thermo, United States). Immediately after the disruption, 10x wash buffer was added to a final concentration of 1x. Cell debris was removed by centrifugation (10000 *g*, 20 min, 4°C) and the supernatant was filtered through a 0.45 μm syringe filter. StrepII-tagged PpiB or CysK were purified from the supernatant using Strep-Tactin^^®^^ sepharose (iba Lifesciences, Germany) according to manufacturer’s instructions. Concentration and buffer change of purified proteins were achieved by centrifugation (5000 *g*, RT) using Vivaspin^^®^^ 20 ultrafiltration units (Sartorius, Germany) with 30 or 10 kDa molecular weight cut offs. Proteins were stored in 100 mM HEPES (pH 7.0) at −20°C. Protein yield and purity were analyzed by SDS-page, and protein concentration was determined by absorption at 280 nm.

### CysK Activity Assay

Enzymatic activity of recombinantly produced CysK of *C. difficile* was measured photometrically as previously described with slight modifications (Tai et al., 1993; Saavedra et al., 2004). Briefly, the assays were performed in a 96-well format in 100 μL final volume. As substrates *O*-Acetyl-L-serine hydrochloride (OAS; Sigma-Aldrich^^®^^, A6262) and 5-Thio-2-nitrobenzoic acid (TNB) were used. 20 mM OAS stock solution was freshly prepared in 100 mM HEPES (pH 7.0). Fresh TNB-stock solution was prepared by dissolving 10 mM 5,5′-Dithiobis(2-nitrobenzoic acid) (DTNB; Sigma-Aldrich^^®^^, D8130) and 15 mM dithiothreitol in 100 mM HEPES (pH 7.0). Each reaction contained 2 mM OAS and 50 μM TNB. Following the addition of CysK at a final concentration of 7 μM, the reactions were monitored in a VarioSkan^TM^ (Thermo Fisher, United States) plate reader at 30°C by measuring the OD_412__nm_ every 4 min. If needed CysK was denatured by incubating aliquots for 30 min at 56°C.

### Determination of Toxin Concentration in Supernatants

For determining the toxin production, exponentially growing cultures were adjusted to an OD_600__nm_ of 0.05 in 10 mL of fresh BHIS and grown at 37°C under anaerobic conditions. After 48 h, the culture supernatants of 2 mL of each culture were harvested (5000 *g*, 5 min, RT) and sterile filtered using 0.2 μm syringe filters. Toxin concentrations in culture supernatants were determined using the *Clostridium difficile* Toxin A OR B ELISA Kit (tgc-E002-1) of tgcBIOMICS (Germany) following the manual instructions.

### Cytotoxicity Assay

In order to visualize the cytotoxic activity of *C. difficile* culture supernatants, NIH-3T3 mouse fibroblast cells (ATCC^^®^^ CRL-1658^TM^, United States) were cultured in DMEM (Gibco, United States) supplemented with 10% FBS at 37°C and 5% CO_2_. For the assay, 10^5^ cells/mL were seeded on glass cover slips in 24-well plates and cultured for 2 days. In parallel, *C. difficile* were grown in anaerobic chamber for 24 h, and the cell free supernatant containing secreted toxins was harvested by centrifugation (8000 *g*, 5 min, RT). This supernatant was diluted to 0.1 % (vol/vol) in cell culture medium. Adherent NIH-3T3 cells were washed once with 1 mL PBS, and 1 mL medium with bacterial culture supernatant was added. After incubation for 2 h, medium was removed and the cells were fixed for 15 min at RT with 2% (wt/vol) PFA in 1x PBS, and subsequently permeabilized with 0.1 % (vol/vol) Triton X-100 in 1x PBS with 1% (wt/vol) BSA for 5 min. Cells were stained with PBS containing 1 μg/mL DAPI (Sigma-Aldrich^^®^^) and 1:1000 diluted Phalloidin-Alexa488 (Abcam, United Kingdom). The mounted samples were analyzed with a confocal microscope (Leica^^®^^ SP8, Germany) using an 63x oil immersion objective.

## Results

### Identification of the Protein Interaction Partners of CdPpiB

**FIGURE 1:**
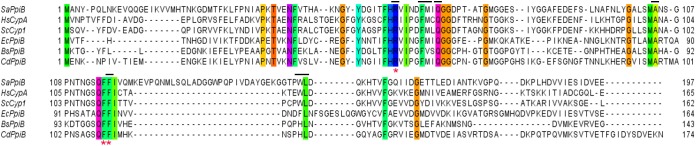
CdPpiB is a classical cyclophilin-type PPIase. The alignment of amino acid sequences of cyclophilin type PPIases of the Gram-positive species *S. aureus* (SaPpiB; Q2FZU9), *C. difficile* (CdPpiB; Q18D70) and *B. subtilis* (BsPpiB; P35137) as well as the Gram-negative model organism *E. coli* (EcPpiB; P23869), and the yeast (ScCyp1; P14832) and human CypA (HsCypA; P62937) reveals highly conserved amino acids along the PPIase active surface (marked by a black line). The alignment was performed using the T-Coffee webserver and visualized by Jalview 2.10.3b1. Amino acids that are conserved to 100% are highlighted by color coding according to Taylor (Taylor, 1986; Notredame et al., 2000; Waterhouse et al., 2009). Amino acids that have been mutated in CdPpiB are marked by an asterisk.

A protein blast search using the PpiB sequence of *Bacillus subtilis* str. 168 (Uniprot ID: P35137) against the most recently annotated *C. difficile* str. 630Δ*erm* genome identified a single potential cyclophilin encoded by the gene locus CDIF630erm_00459 (CD630_00330 in *C. difficile* 630). This gene is flanked upstream by a gene potentially encoding for a patatin-like phospholipase, and downstream by a gene encoding the putative exosporium glycoprotein BclA1 (Dannheim et al., 2017a). An alignment of the potential *C. difficile* cyclophilin with bacterial cyclophilins of *E. coli* str. K12 (EcPpiB; P23869), *B. subtilis* (BsPpiB; P35137) and *Staphylococcus aureus* str. NCTC8325 (SaPpiB; Q2FZU9) as well as eukaryotic representative members of yeast (ScCyp1; P14832) and human (HsCypA; P62937) revealed high amino acid sequence similarities between the different proteins. An exceptional high degree of conservation was observed for amino acid residues involved in PPIase activity (Figure 1). Accordingly, this gene was selected for destruction by ClosTron, which resulted in the generation of a Δ*ppiB* mutant in the *C. difficile* str. 630Δ*erm* background.

**FIGURE 2:**
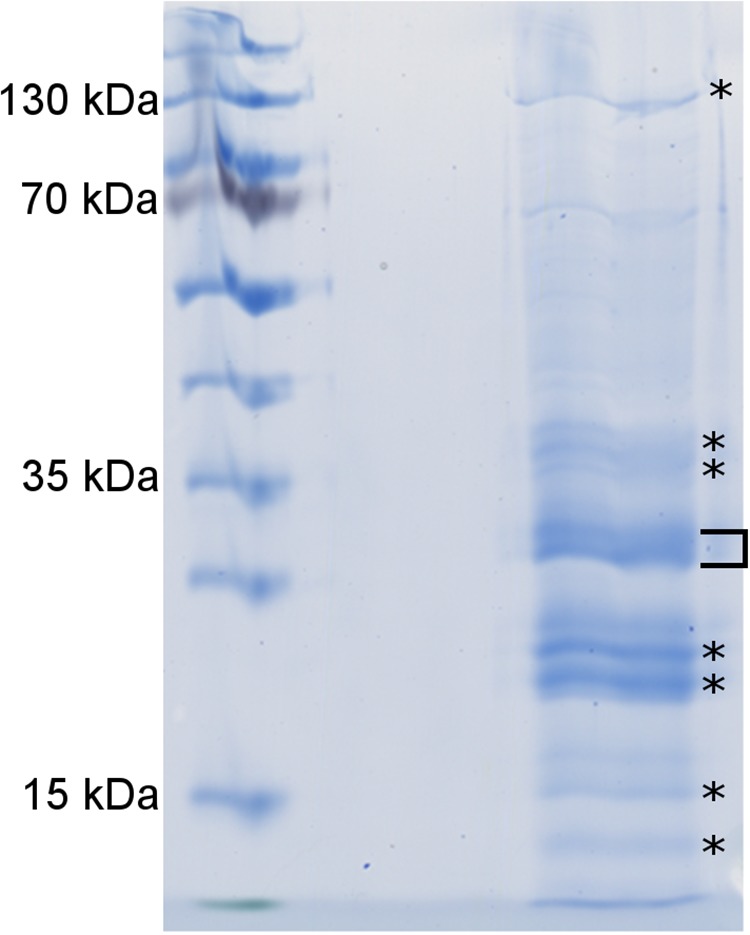
*In vivo* cross-linking reveals putative interaction partners of CdPpiB. Shown is a representative analytical SDS-PAGE with the elution fraction after the *in vivo* cross-linking and the subsequent Strep-tag affinity purification of PpiB-StrepII and its putative interaction partners. Bands denoted with an asterisk were cut out analyzed by mass spectrometry. The bracket indicates bands that also occurred in control experiments without cross-linking and were excluded from the analysis.

Next, we identified *in vivo* protein interaction partners of CdPpiB using an interactomics approach by using N-terminally StrepII-tagged PpiB as bait and formaldehyde as a cross-linker. A preparative SDS-PAGE of the purified protein complexes revealed in total, seven distinct protein bands that were cut out and prepared for LC-MS analysis (Figure 2).

This yielded 39 unique hits, and as expected, PpiB was found to be with 83% the best covered protein among those. The majority of the remaining 38 hits that resembled proteins interacting either directly or indirectly in larger complexes with CdPpiB belonged to the functional groups metabolism (19 hits, 50%) and stress response (8 hits, 21%). The rest of the hits were distributed among transcription/translation, protein folding and transport (3 hits and 8% each) as well as motility/surface (2 hits 5%) (Table 3). Among the retrieved potential interaction partners were LdhA, AcdB, HadA, EtfA1, EtfA3, and EtfB1. These enzymes represent the complete pathway for phenylalanine and leucine fermentation (Kim et al., 2006). Similarly, the also detected pyruvate carboxylase Pyc, pyruvate-ferredoxin oxidoreductase Pfo, aldehyde-alcohol dehydrogenase AdhE2 and pyruvate kinase Pyk as well as the glyceraldehyde-3-phosphate GapA and fructose-1,6-bisphosphate aldolase Fba are central enzymes of carbon metabolism (Dannheim et al., 2017b). Finally, the *O*-acetylserine sulfhydrylase/cysteine synthase (CysK), was among the metabolic proteins the one with the highest sequence coverage. It represents the last enzyme of cysteine biosynthesis (Dubois et al., 2016).

**Table 3:** Putative interaction partners of CdPpiB identified by *in vivo* cross-linking.

Class	Name	Accession-Nr.^†^	Gene locus^†^	Coverage (%)	# unique peptides	Function
Metabolism	CysK	ARE62524.1	01767	40	13	Cysteine synthase activity
	GapA	ARE64122.1	03466	36	14	Glyceraldehyde-3-phosphate dehydrogenase
	Fba	ARE61306.1	00531	34	11	Fructose-1,6-bisphosphate aldolase
	Pyc	ARE60920.1	00083	32	40	Pyruvate carboxylase
	EtfA1	ARE61304.1	00529	28	10	Isocaproyl-CoA dehydrogenase, electron transfer flavoprotein alpha subunit
	AcdB	ARE61302.1	00527	24	8	Isocaproyl-CoA dehydrogenase, catalytic subunit
	PduL	ARE63606.1	02937	24	5	Phosphate propanoyltransferase
	Putative NUDIX-family hydrolase	ARE61693.1	00909	24	3	Hydrolase
	Pfo	ARE63605.1	02936	23	25	Pyruvate synthase
	NifU-like protein	ARE62199.1	01433	23	3	[Fe–S]-cluster assembly protein
	AdhE2	ARE63909.1	03250	20	17	Aldehyde-alcohol dehydrogenase
	LdhA	ARE61297.1	00522	20	6	D-lactate dehydrogenase
	PfkA	ARE64384.1	03700	20	4	ATP-dependent 6-phosphofructokinase
	EtfA3	ARE61965.1	01196	19	6	Electron transfer flavoprotein subunit alpha
	Putative 5-nitroimidazole reductase	ARE62385.1	01623	19	3	Putative 5-nitroimidazole antibiotic resistance protein
	HadA	ARE61298.1	00523	18	6	Isocaprenoyl-CoA:2-hydroxyisocaproate CoA-transferase
	IorB	ARE63301.1	02620	18	3	Indolepyruvate oxidoreductase, subunit beta
	EtfB1	ARE61303.1	00528	17	4	isocaproyl-CoA dehydrogenase, electron transfer flavoprotein beta subunit
	Pyk	ARE64383.1	03699	16	7	Pyuvate kinase
Stress Response	Rbr1	ARE61727.1	00944	58	11	Iron ion binding, oxidoreductase activity, rubrerythrin
	Dsr	ARE61729.1	00946	36	4	*In silico* predicted desulfoferrodoxin
	Rbr2	ARE62400.1	01638	35	6	Iron ion binding, oxidoreductase activity, rubrerythrin
	Rbr3	ARE62452.1	01692	33	6	*In silico* predicted reverse rubrerythrin
	UspA	ARE61714.1	00930	22	3	Putative universal stress protein A
	YloU	ARE63487.1	02813	20	2	Putative alkaline-shock protein
	Alkaline shock protein (YqhY)	ARE62115.1	01349	17	2	Alkaline shock protein
	CspB	ARE62276.1	01511	15	2	Cold shock protein
Transcription/ Translation	Lrp	ARE64537.1	03859	30	4	AsnC-family transcriptional regulator
	InfC	ARE61586.1	00798	17	2	Translation initiation factor IF-3
	RFF	ARE63058.1	02368	16	2	Ribosome-recycling factor
Protein folding	Putative phage protein	ARE61884.1	01109	29	5	Protein-export protein, SecB-like
	GroS	ARE61098.1	00314	23	2	10 kDa chaperonin
	DnaK	ARE63387.1	02707	22	13	Chaperone protein, unfolded protein response
Transport	ABC transporter substrate- binding protein	ARE61772.1	00993	34	8	ABC-type transport system, sugar-family extracellular solute-binding protein
	Biotin carboxyl carrier protein	ARE62848.1	02143	23	5	*In silico* predicted biotin carboxyl carrier protein
	UPF0145 protein	ARE62649.1	01899	23	2	Putative heavy metal binding
Motility/Surface	FliC	ARE61143.1	00361	54	19	Flagellin
	SlpA	ARE63719.1	03056	19	11	S-layer precursor protein

*^†^as annotated in str. 630Δerm by Dannheim et al. (2017a) (GenBank: CP016318.1).*

A whole battery of stress response proteins was also found attached to CdPpiB, including the two rubyerythrins Rbr1 and Rbr2 and the desulfoferrodoxin Dsr, which are involved in oxidative stress response (Kawasaki et al., 2009; Riebe et al., 2009). Furthermore, the cold shock protein CspB and the two alkaline shock proteins YloU and YqhY as well as the universal stress protein UspA were among the interacting proteins (Nyström and Neidhardt, 1993; Söderholm et al., 2011; Derman et al., 2015; Tödter et al., 2017). The chaperones GroS and DnaK are part of multiple stress responses (LaRossa and Van Dyk, 1991). Key players of translational quality control, the ribosome-recycling factor RRF, translation initiation factor IF-3 InfC and most interestingly, the pleiotropic transcriptional regulator Lrp were further proteins associated with CdPpiB (Brinkman et al., 2003; Ohashi et al., 2003; Laursen et al., 2005). The screen also yielded flagellin (FliC, 54% coverage) and surface layer protein (SlpA, 19% coverage), two major virulence associated proteins (Tasteyre et al., 2001; Merrigan et al., 2013). Finally, a sugar-family solute binding protein, a putative heavy metal binding protein (UPF0145) and a predicted biotin carrier protein were identified as transporter or transporter associated proteins cross-linked to CdPpiB (Table 3).

### CdPpiB Interacts With Lrp, FliC and CysK in a Bacterial Two Hybrid System

**FIGURE 3:**
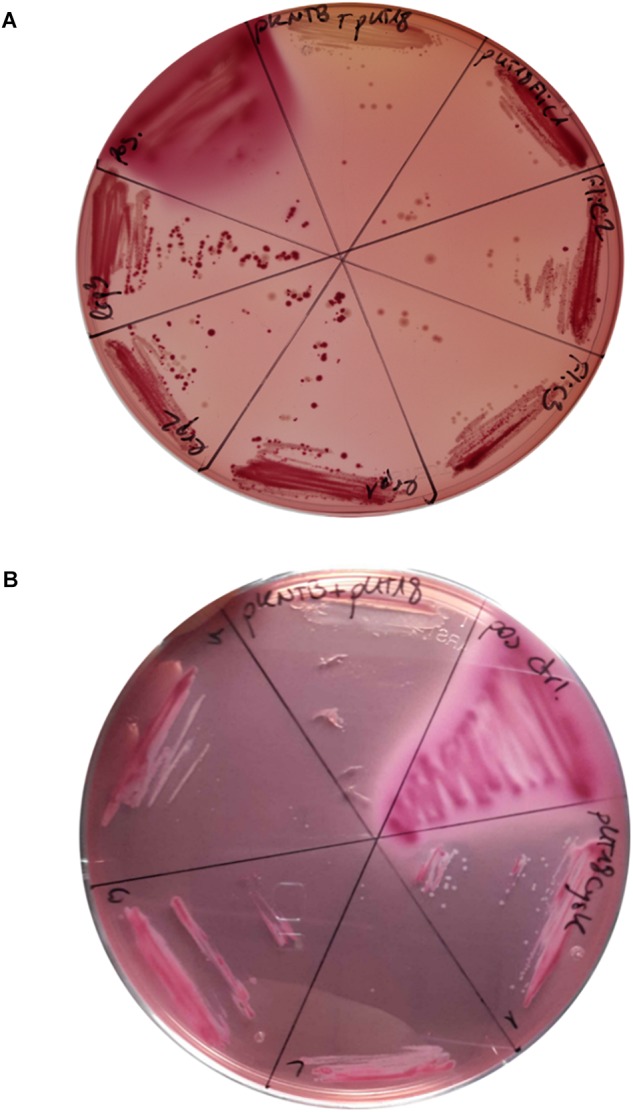
Confirmation CdPpiB interaction partners with BACTH on MycKonkey-Agar. **(A)** *E. coli* BTH101 cells transformed with wt *ppiB* carrying pKNTB in combination with pUT18_FliC carrying wt *fliC* or pUT18_Lrp carrying wt *lrp* were streaked out on MacKonkey-agar and grown for 2 days at 30°C. **(B)** *E. coli* BTH101 cells transformed with wt *ppiB* carrying pKNTB in combination with pUT18_CysK carrying wt *cysK* were streaked out on MacKonkey-agar and grown for 2 days at 30°C. Purple coloring was indicative of protein interaction due to adenylate cyclase activity. On each plate *E. coli* BTH101 carrying empty pUT18 plasmid in combination with pKNTB served as negative control, whereas colonies carrying the pUT18-zip and pKT25-zip plasmids delivered by the manufacturer served as positive control. At least three colonies of each transformation were tested. Representative plates of three separate transformations are shown.

In order to verify direct interactions between candidate proteins identified by our interactomic studies, we applied bacterial two hybrid (BACTH) based protein-protein interaction tests. For this, we chose Lrp as it is a global transcriptional regulator, and as there are no reports on functional interactions between bacterial PPIases and transcriptional regulators. Another candidate was the metabolic enzyme with the highest coverage, CysK, that participates in cysteine metabolism which is central to virulence of *C. difficile* (Dubois et al., 2016). FliC was chosen as it is a well-established virulence factor in many pathogenic bacteria including *C. difficile* (Tasteyre et al., 2001). For all the tested proteins a clear-cut interaction with CdPpiB was observed, indicated by pink coloring of the resulting clones on MacConkey-agar verifying our findings from the interactomics study (Figure 3).

### An Intact PPIase Domain of CdPpiB Is Required for the Interaction With P87 Substrate of Lrp

**FIGURE 4:**
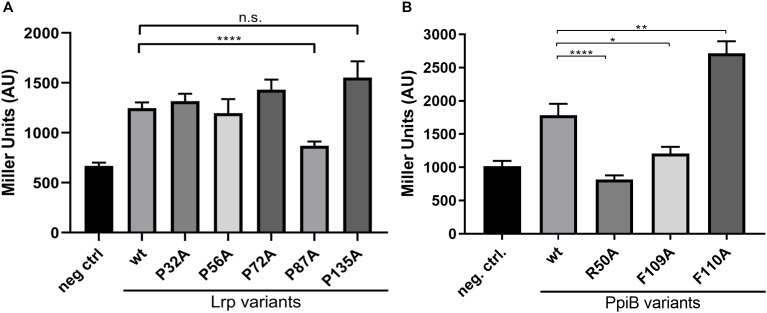
CdPpiB targets P87 in CdLrp and this interaction depends on conserved amino acids. **(A)** β-galactosidase activities of *E. coli* BTH101 clones carrying combinations of wt CdPpiB and single proline exchange mutants of CdLrp. **(B)** β-galactosidase activities of *E. coli* BTH101 clones carrying combinations of wt CdLrp and single amino acid exchange mutants of CdPpiB. Clones carrying wt CdPpiB and the empty companion vector pKNT25 served as negative control in both experiments. Shown are mean and SEM of three independent experiments with at least five clones each. Statistical significance was calculated by unpaired *t*-Test with Welch’s correction (^∗^*p* ≤ 0.05, ^∗∗^*p* ≤ 0.01, ^∗∗∗∗^*p* ≤ 0.0001, n.s., not significant).

Following the confirmation of the interaction via the BACTH screen, we wanted to analyze whether these interactions may be dependent on the PPIase activity of CdPpiB. For this purpose, we chose Lrp for a detailed study as it is with 15 kDa comparably small and has only five proline residues. As PPIases act on the peptidyl-prolyl bonds in proteins, we exchanged all five proline residues in Lrp by alanine generating the single amino acid substitution mutants P32A, P56A, P72A, P87A and P135A. The interactions between CdPpiB and the generated Lrp-variants were quantified in β-galactosidase assays using the outlined BACTH system. Among the five Lrp-variants only variant P87A showed a significantly reduced β-galactosidase activity. It was with 868.8 ± 43.55 MU 30% less than the interaction with wild type Lrp which had an average β-galactosidase activity of 1246 ± 59.06 MU indicating a specific interaction between the two proteins (Figure 4A). In order to prove that the PPIase domain is involved in the overall process, we went on by exchanging highly conserved single amino acid residues within the active site of CdPpiB that are known from homologous cyclophilins of Gram-positive and Gram-negative bacteria (Göthel et al., 1996; Skagia et al., 2017a; Wiemels et al., 2017). Accordingly, we generated the CdPpiB variants R50A, F109A and F110A (s.a. Figure 1). For all three mutants, significant differences were observed regarding the interaction with Lrp as assessed by β-galactosidase activity (Figure 4B).

### Destruction of *ppiB* Leads to Sensitivity to Envelope Stress and L-Cysteine in *C. difficile*

**FIGURE 5:**
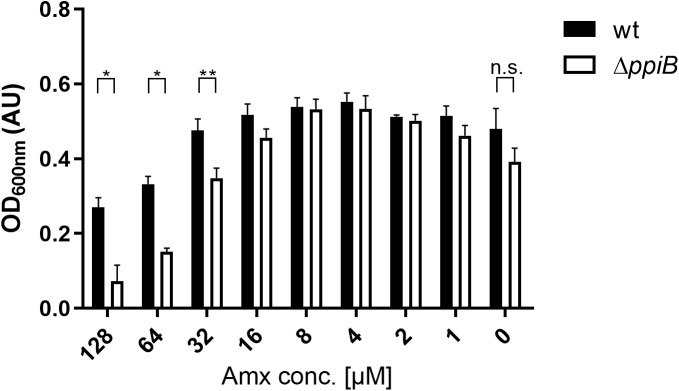
CdPpiB-deficiency increases susceptibility toward amoxicillin. Wild type and its isogenic Δ*ppiB*-mutant were grown in the presence of decreasing concentrations of amoxicillin (Amx) and bacterial growth was measured at 600 nm. The graph depicts mean ± SEM of three separate experiments performed in duplicates. Statistical significance was calculated by unpaired *t*-Test (^∗^*p* ≤ 0.05, ^∗∗^*p* ≤ 0.01, n.s., not significant).

As our interactomic data revealed that CdPpiB interacts with several stress response proteins, we performed a literature survey of available transcriptomic data. By this, we found that *ppiB* was the only PPIase of *C. difficile* that was significantly upregulated under cell wall stress caused by the beta-lactam amoxicillin (Emerson et al., 2008). Accordingly, we tested the susceptibility of the Δ*ppiB* mutant in a serial dilution assay, and registered that the mutant was significantly more susceptible to amoxicillin concentrations ≥ 32 μM supporting our findings regarding the involvement of CdPpiB in stress tolerance (Figure 5).

**FIGURE 6:**
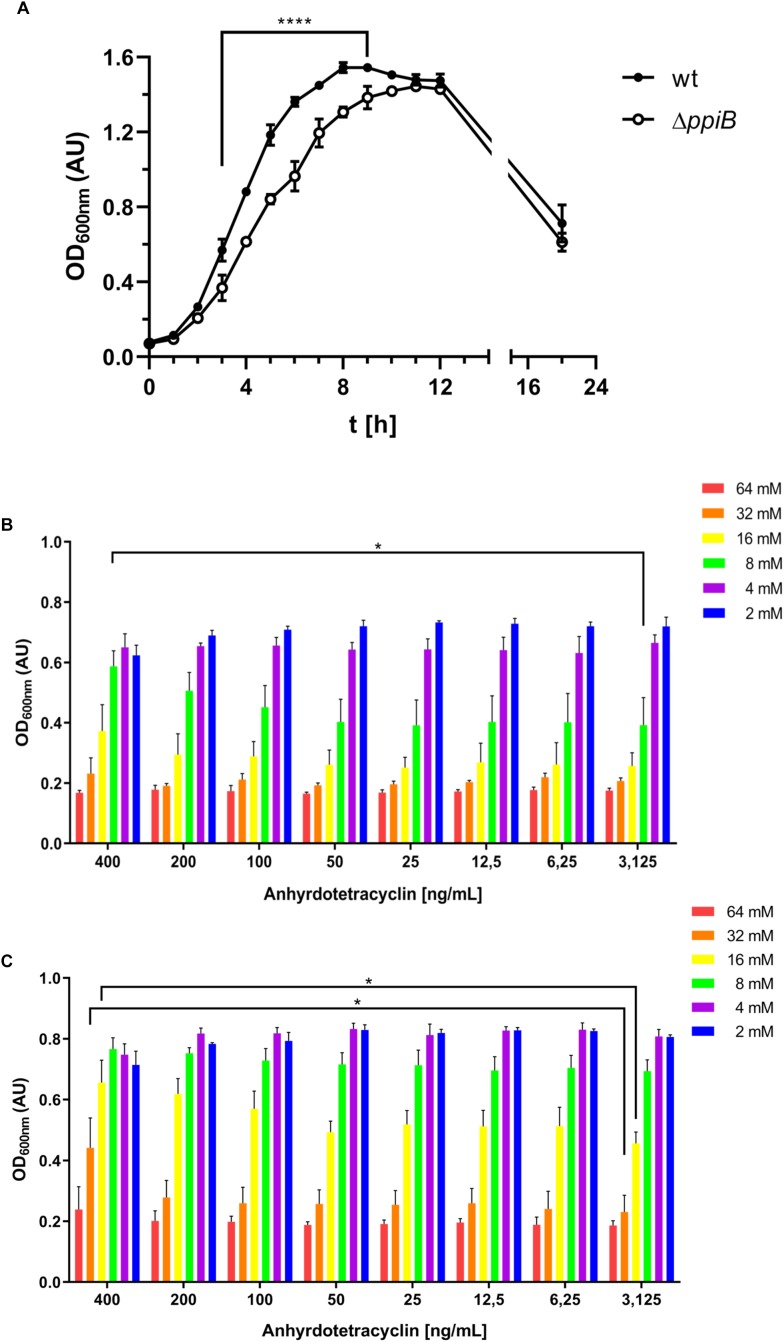
CdPpiB confers cysteine tolerance to *C. difficile*. **(A)** Destruction of *ppiB* causes growth defect in BHIS as assessed by the change in the OD_600__nm_ over time. **(B)** Complementation of the Δ*ppiB* mutant through induction with 400 ng/mL anhydrotetracycline allows the bacteria to cope with 8 mM L-cysteine when grown in BHI supplemented with different concentrations of cysteine. **(C)** Stepwise over-expression of *ppiB* with increasing amounts of anhydrotetracycline raises the tolerance of the wild type toward 16 and 32 mM L-cysteine. Shown are means ± SEM of three independent experiments performed in duplicate. Statistical significance was calculated by unpaired *t*-Test (^∗^*p* ≤ 0.05, ^∗∗∗∗^*p* ≤ 0.0001).

We further went on characterizing the generated Δ*ppiB* mutant by performing growth assays using the standard rich medium BHIS, which is supplemented with 0.5% (wt/vol) yeast extract and 0.1% (wt/vol) L-cysteine. Here, the Δ*ppiB* mutant displayed a significantly delayed growth in BHIS medium containing L-cysteine. Slower growth in the exponential phase resulting in a later entry into stationary phase (9 h in wt vs. 12 h in Δ*ppiB*) was observed. The final OD after 22 h, were comparable for both strains (Figure 6A). When the assays were repeated with plain BHI or with BHIS lacking L-cysteine, no difference between wild type and the Δ*ppiB* mutant were observed. These tests identified L-cysteine as the cause for the observed phenotype. In order to verify the *ppiB* mutation as sole cause for the observed differences, we complemented the mutant with an intact *ppiB* gene *in trans* and brought *ppiB* under the control of the tetracycline promoter (P_tet_) allowing fine-tuned expression under the control of anhydrotetracycline (aTc). The wild type strain and its isogenic Δ*ppiB* mutant were transformed, and grown in the presence of different concentrations of L-cysteine (2–62 mM) in combination of increasing concentrations of aTc (3,625-400 ng/mL) (Figure 6B,C). When fully induced with 400 ng/mL, the Δ*ppiB* mutant was able to cope with 8 mM L-cysteine, which equals to the concentration in BHIS. Its growth under full induction was significantly better compared to reduced induction with 200 ng/mL or less aTc (Figure 6B). Overexpressing *ppiB* in the wild type resulted in an even higher tolerance of up to 32 mM L-cysteine and a significantly better growth compared to lower induction levels. In case of 16 mM L-cysteine the wild type overexpressing *ppiB* reached optical densities comparable to the wild type gown at 8 mM L-cysteine without *ppiB* overexpression (Figure 6C). These observations corroborated the interactomic data where CysK was the major metabolic protein interacting with CdPpiB.

### CdPpiB Restores CysK Activity After Heat Inactivation

**FIGURE 7:**
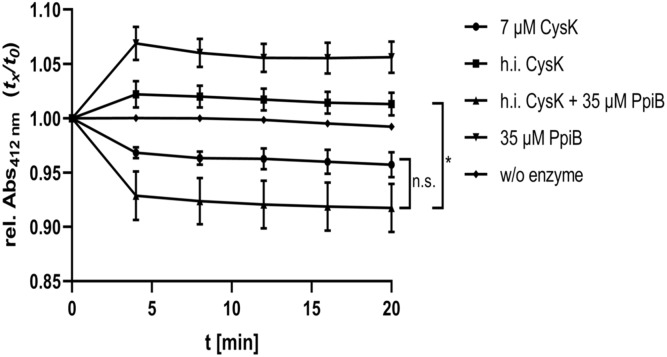
CdPpiB restores CysK-activity after heat denaturation. *O*-acetyl-sulfhydrylase activity of recombinant CysK was measured by the decrease of OD_412_ at 30°C. Activity of 7 μM recombinant CysK diminished after inactivation at 56°C for 30 min, and could be restored by the addition of recombinant PpiB in 5-times molar excess. Shown are mean and SEM of three independent measurements proteins of two different productions. Statistical significance was calculated by unpaired *t*-Test with Welch’s correction (^∗^*p* ≤ 0.05, n.s., not significant).

One important function of PPIases is the restoration of enzyme activity after protein inactivation by unfavorable chemical or physical conditions. In our interactomic study, we identified and verified CysK, the terminal enzyme in L-cysteine biosynthesis, as an interaction partner of CdPpiB. Accordingly, we tested the influence CdPpiB on heat inactivated CysK, the last enzyme of cysteine biosynthesis. Activity of recombinantly produced CysK was measured in a photometric assay at 412 nm, and was shown to be active at 7 μM concentration. Next, CysK was mildly heat denatured at 56°C for 30 min and its enzymatic activity was measured with or without five-fold excess of CdPpiB. As expected, heat denatured CysK showed no activity. But, the addition of 35 μM CdPpiB to the reaction efficiently restored its enzymatic activity (Figure 7). This indicates that CdPpiB functionally interacts with CysK and promotes its structural stability.

### Deletion of *ppiB* Increases Toxin Production and Cytotoxic Activity

**FIGURE 8:**
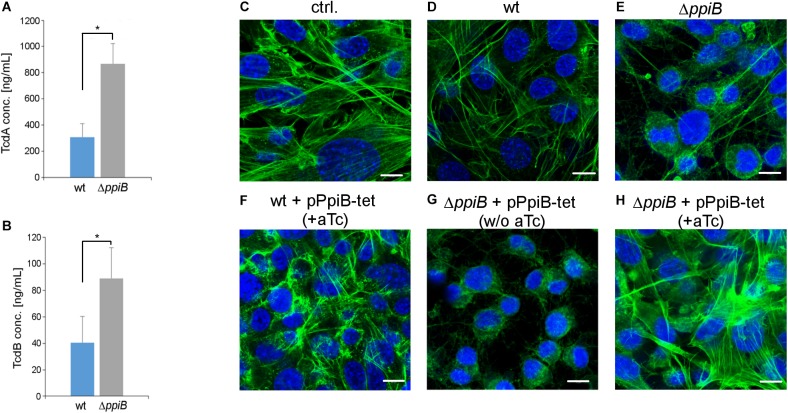
Deletion of *ppiB* leads to higher toxin titres and cytotoxicity. **(A)** TcdA and **(B)** TcdB concentrations were measured by ELISA in 48 h old culture supernatants. Both toxins accumulated to significantly higher titres in culture supernatants in the isogenic Δ*ppiB* mutant. Shown are means ± SD of three independent experiments performed in duplicate. Statistical significance was calculated by unpaired *t*-Test (^∗^*p* ≤ 0.05). **(C–H)** PpiB influences the cytotoxic activity in the culture supernatant. **(C)** In untreated control NIH-3T3 cells the actin cytoskeleton is organized in stressfibers as it is typical for epithelial cells. **(D)** Cell-free supernatants from 24 h old wild type cultures that were diluted to 0.1% (vol/vol) in cell culture medium have visually no detrimental effect on the actin cytoskeleton. **(E)** Supernatants of the Δ*ppiB* mutant cause disintegration and rounding up of the cells at the same dilution. **(F)** Overexpression of *ppiB* by the addition of 400 ng/mL anhydrotetracycline (aTc) in a tetracycline-inducible system has no detrimental effect on the cells in the wild type background. **(G)** The complemented strain exerts comparable cytotoxic activity as the Δ*ppiB* mutant in the absence of aTc. **(H)** Overexpression of *ppiB* by the addition of aTc reduces the cytotoxic effect in the Δ*ppiB* background to wild type levels. Shown are representative views of two separate experiments performed in triplicates. Actin cytoskeleton was stained green with Alexa488-coupled phalloidin, while nuclear DNA was stained blue with DAPI. Scale bars correspond to 10 μm.

Considering that CysK is a central enzyme in L-cysteine metabolism and that this amino acid is also a potent suppressor of toxin production, we tested the influence of CdPpiB on the cellular amounts of TcdA and TcdB under *in vitro* conditions (Karlsson et al., 2000; Dubois et al., 2016). We quantified both toxins in stationary cultures of wild type and the *ppiB* mutant using ELISA. Both toxins were found in significantly higher concentrations in the culture supernatants of the *ppiB* mutant. While the wild type strain revealed in average 309 ng/mL TcdA in its culture supernatant, the *ppiB* mutant produced 2.8-fold more TcdA (868 ng/mL) (Figure 8A). Similarly, TcdB was 2.3-fold higher concentrated in the growth supernatant of the mutant (89 ng/mL) compared to the wild type (40 ng/mL) (Figure 8B). In accordance with the amounts of toxins found in the culture supernatant, the broth from mutant growth was considerably more active at 0.1% (vol/vol) dilution, causing stronger actin depolymerization and cell rounding of NIH-3T3 cells (Figure 8C–E). Similar results were obtained for the mutant complemented with the *ppiB* gene *in trans*, however, without gene induction by aTc (Figure 8G). Wild type levels of toxins and corresponding cytotoxic effect similar to wild type were observed when the complemented mutant was induced with 400 ng/mL aTc (Figure 8H). Cells treated with supernatants from wild type bacteria overexpressing *ppiB* exhibited no visual abnormalities of the cellular morphology (Figure 8F). Hence, CdPpiB not only interacts with components of the L-cysteine metabolism and supports the growth in the presence of L-cysteine, but it also affects toxin production in *C. difficile*.

## Discussion

The aim of this study was to gain insights into the physiological and virulence related functions of the cyclophilin CdPpiB of the nosocomial pathogen *C. difficile*. Our *in vivo* interactomics approach in combination with bacterial two hybrid testing allowed us the identification and verification of a specific set of interaction partners that are major control proteins of cell physiology and virulence. The majority of the interaction partners belonged to the category of energy and central metabolism (Table 3). Here, several functionally related proteins were identified, including LdhA, EtfA1, AcdB, EtfA3, HadA, and EtfB1. These enzymes are part of the same Stickland reaction involving L-leucine catabolism. By this, *C. difficile* harnesses metabolic energy from the oxidation of one equivalent of L-leucine to isovalerate, CO_2_ and NH_4_^+^. In return, two equivalents of L-leucine are reduced to isocapronate and NH_4_^+^ (Stickland, 1934; Britz and Wilkinson, 1982; Kim et al., 2006). This pathway is coupled via NAD^+^ and ferredoxin to the RNF complex responsible for proton/sodium gradient formation required for ATP generation in *C. difficile* (Kim et al., 2004; Aboulnaga et al., 2013).

Another group of proteins of putative interaction partners belonged to the primary carbon metabolism. These included pyruvate carboxylase Pyc, pyruvate-ferredoxin oxidoreductase Pfo, aldehyde-alcohol dehydrogenase AdhE2 and pyruvate kinase, which control the flux around the pyruvate knot of the central metabolism. On the other hand, the glyceraldehyde-3-phosphate GapA and fructose-1,6-bisphosphate aldolase represent key control points of glycolysis (Dannheim et al., 2017b). Interestingly, *gapA* and *adhE2* were also up-regulated under cysteine rich conditions (Dubois et al., 2016; Gu et al., 2018). Thus, CdPpiB potentially controls the major fluxes of the central metabolism of *C. difficile*.

The second largest group of interaction partners belonged to the group of stress response, including proteins protecting against temperature, oxidative and alkaline shock. In line with this, the Δ*ppiB* mutant was more susceptible to amoxicillin, which causes cell wall stress. This finding was also in accordance with a previous microarray study, where *ppiB* was significantly upregulated in amoxicillin treated *C. difficile* (Emerson et al., 2008). Currently, we do not know how CdPpiB may influence this phenotype. However, the well-known chaperones DnaK and GroS as well as YloU and YqhY were also upregulated in case of amoxicillin stress (Emerson et al., 2008). In *S. aureus* DnaK is part of the cell wall stress stimulon, and *dnaK* mutants have a higher susceptibility toward the beta-lactam oxacillin (Pechous et al., 2004; Singh et al., 2007). Recently, DnaK was identified as a binding partner of EcPpiB in functional complementation and co-precipitation studies in *E. coli*, where it positively influenced the enzymatic activity and correct localization of DnaK (Skagia et al., 2016). Hence, it is conceivable that, also in *C. difficile*, CdPpiB is part of the DnaK stress response network. This feature might further be supported by the interactions with YloU and YqhY, which are close homologs of the alkaline shock protein 23 (Asp23) of *S. aureus* that is also associated with cell envelope stress (Müller et al., 2014; Tödter et al., 2017). EcPpiB is connected to another cell envelope related phenomenon, namely, cell division by directly interacting with FtsZ and probably further client proteins (Skagia et al., 2017b). Our interactomic study did not identify FtsZ as interaction partner in *C. difficile*. This might indicate that cyclophilins perform species-specific duties despite their high degree of conservation.

Several oxidative stress response proteins of the group of (Rbr1, Rbr2, and Rbr3) and a desulfoferrodoxin (Dsr) were found to bind to CdPpiB. Rubrerythrins are small proteins that have NADH-dependent peroxidase activity and act in concert with rubredoxins and desulfoferrodoxins (Mishra and Imlay, 2012). The homologs of Rbr1 and Dsr are important for conferring O_2_-tolerance to *C. acetbutylicum* under the control of the transcriptional regulator PerR, which located between these two genes in *C. difficile* (Hillmann et al., 2008; Kawasaki et al., 2009; Dannheim et al., 2017a). As these proteins act in close vicinity for efficient electron transfer, it is likely that CdPpiB is involved in complex formation or stability supporting their physiological function. Moreover, oxidative stress genes are upregulated in the presence of L-cysteine in *C. difficile* (Dubois et al., 2016; Gu et al., 2018).

Two well-established virulence partners, namely the flagellar subunit FliC and the surface layer protein SlpA, were among the CdPpiB interaction partners. In case of FliC this interaction was confirmed by BACTH indicating a functional relationship between these proteins. Two oligomeric states of FliC seem to co-exist: the monomer which has been found in diverse cellular compartments and the polymeric organelle which is employed for motility, attachment and penetration (Campodónico et al., 2010). In fact, the implications of FliC monomers in invasiveness and virulence to the host are not restrained to *C. difficile* but apply to many other bacteria. For instance, in *Pseudomonas aeruginosa* or *Salmonella typhimurium* FliC appears to be the most prominent virulence factor in terms of inflammasome and immune response activation (Duncan and Canna, 2018; Garcia et al., 2018). Interestingly, FliC was shown to interact with DnaK in the periplasm of *P. aeruginosa*, and in *C. difficile* a Δ*dnaK* mutant had four-fold diminished *fliC* and four-fold increased *groS* expression (Borrero-de Acuña et al., 2016; Jain et al., 2017). This supports our idea of CdPpiB being a crucial part of the chaperone network of *C. difficile*.

Next to some transport related proteins, very crucial factors of translational fidelity, IF-3 and RRF, were detected by interactomics. IF-3 is involved in the stabilization of the 30S ribosomal subunit, enabling mRNA binding to the 30S subunit and warranting the accuracy of the first aminoacyl-tRNA binding (Hua and Raleigh, 1998). The RRF, on the other hand, facilitates the dissociation of ribosomes from mRNA after termination of translation. RRF was together with elongation factor P one of the two highly upregulated translation factors in *B. subtilis* (Ohashi et al., 2003). As a foldase CdPpiB might, indeed, be in close vicinity of ribosomes and assist folding of newly synthesized proteins. However, there are currently no reports supporting this. Also, there is with trigger factor a known ribosome associated protein with a FKBP-domain that was shown to accompany proteins during their maturation following translation (Göthel et al., 1998; Kawagoe et al., 2018). This ubiquitous protein is also present in *C. difficile* (CDIF630erm_03607/CD630_33060) and is accordingly expected to be the major PPIase that facilitates protein folding. Nevertheless, it could be that CdPpiB does not associate with newly synthesized proteins but is involved in the recycling of translation factors for efficient ribosomal fidelity.

One of the most interesting binding partners was the transcriptional regulator Lrp. Hence, we analyzed the putative interaction between CdPpiB and Lrp in more detail and showed that this interaction depends on the PPIase active site residues. Mutating the highly conserved R50 and F109 weakened, while a F110A substitution significantly strengthened the association between both proteins. In return, out of the five proline residues of CdLrp only mutating P87 reduced the interaction of both proteins indicating a specific PPIase client protein relationship. Lrp-type proteins are global transcriptional regulators that consist of a classical N-terminal helix-turn-helix DNA-binding domain and a C-terminal substrate binding/ activation domain and typically act as specific regulators of amino acid metabolism-related genes (Brinkman et al., 2003). However, in *B. subtilis* LrpC, a close homolog of CdLrp, was also shown to be important for DNA-repair (López-Torrejón et al., 2006). More interestingly, deleting *decR* (STM0459), a very close homolog of CdLrp in S*almonella typhimurium*, rendered the bacteria more susceptible to L-cysteine (Oguri et al., 2012). Hence, the cysteine-dependent phenotypes of the Δ*ppiB* mutant might be due to the participation of CdPpiB on multiple levels ranging from gene regulation to enzymatic activity. The proline residue at position 87 is in the vicinity of the substrate binding/activation domain. Considering that peptidyl-prolyl-*cis/trans*-isomerization results in conformational changes in proteins, it is possible that CdPpiB assists CdLrp in changing its conformation upon binding its substrate or that it regulates the conformational equilibrium of CdLrp for a fine-tuned gene expression. Currently, only for eukaryotic PPIases examples of gene regulatory actions in the form of transcription complex stabilization or mRNA maturation are known (Hanes, 2015; Thapar, 2015). To the best of our knowledge, this is the first time a direct interaction between a PPIase and a transcriptional regulator with direct implications on gene regulation has been shown in bacteria.

Interestingly and also in accordance with the interactomic findings, the Δ*ppiB* mutant showed two L-cysteine dependent phenotypes: a higher susceptibility toward L-cysteine and accumulation of more toxin in the supernatant. Typically added to the medium to promote growth, L-cysteine has also some detrimental effects because of the generated H_2_S during its degradation. As a result, deleting cysteine catabolizing genes results in decreased cysteine tolerance in bacteria (Oguri et al., 2012; Gu et al., 2017). Cysteine is also among several metabolic stimuli that inhibit toxin production in *C. difficile* on transcriptional level, and it does so via its degradation byproducts pyruvate and H_2_S (Karlsson et al., 2000; Dubois et al., 2016). That cysteine tolerance and regulation of toxin expression converges via the cysteine metabolism was recently shown in a mutant lacking the cysteine desulfhydrase CdsB. This mutant was impaired in its growth in the presence of 5 mM L-cysteine and its toxin production was unaffected by L-cysteine (Gu et al., 2017). These observations overlap with the Δ*ppiB* mutant in our study, and are further supported by the identification of CysK as an interaction partner of CdPpiB. As a *O*-acetylserine sulfhydrylase CysK catalyzes the synthesis of L-cysteine from *O*-acetyl-L-serine and sulfide (Kredich et al., 1979). But it was also speculated that at higher L-cysteine concentrations CysK catalyzes the opposite reaction and contributes to L-cysteine degradation by desulfhydration (Auger et al., 2005; Awano et al., 2005; Dubois et al., 2016). This was further supported by the observation that in *C. difficile* CysK was together with CysE one of the two transcriptionally most up-regulated genes of the cysteine metabolism in L-cysteine treated *C. difficile* (Dubois et al., 2016; Gu et al., 2018). In our study, we showed that CdPpiB interacts with CysK and it restores the enzymatic activity of denatured CysK. Considering this and the similarities between the phenotypes, we propose that CdPpiB contributes to L-cysteine tolerance of *C. difficile* by stabilizing the metabolic network on the enzymatic level. Here again, a cooperative action with other chaperones cannot be excluded as *dnaK* and *groS* were also among the highly up-regulated genes in the presence of L-cysteine (Dubois et al., 2016). Furthermore, a direct or indirect influence on the transcription of toxin genes by CdPpiB cannot be excluded and would need further analysis.

Taken together, CdPpiB is a key regulator that acts at the protein folding and modification level and controls central cellular processes. We also suggest that targeting CdPpiB as a therapeutic strategy aiming at virulence reduction may prove intricate, since in the absence of PpiB the bacteria become more toxigenic. Our findings confirm the contribution of the PPIase domain to its regulatory activity. But recent findings also suggest that cyclophilin function in bacteria does not solely rely on PPIase activity (Skagia et al., 2017b; Keogh et al., 2018). Accordingly, whether the PPIase-activity itself or some domains other than the PPIase domain are crucial for a certain interaction or even biological function still need to be evaluated in the future.

## Author Contributions

CÜ, JBdA, LJ, DJ, and MS conceived the experiments. CÜ, MK, MB, and CP conducted the experiments. JW performed the MS/LC analysis. CÜ, MK, MB, LJ, DJ, and MS analyzed the data. CÜ, DJ, and MS drafted and finalized the manuscript. All authors reviewed and approved the final manuscript.

## Conflict of Interest Statement

The authors declare that the research was conducted in the absence of any commercial or financial relationships that could be construed as a potential conflict of interest.

## Footnotes

1 http://www.clostron.com/
